# The novel surfactant protein SP-H enhances the phagocytosis efficiency of macrophage-like cell lines U937 and MH-S

**DOI:** 10.1186/1756-0500-7-851

**Published:** 2014-11-26

**Authors:** Ebru Diler, Martin Schicht, Andrea Rabung, Thomas Tschernig, Carola Meier, Felix Rausch, Fabian Garreis, Lars Bräuer, Friedrich Paulsen

**Affiliations:** Institute of Anatomy and Cell Biology, Saarland University, 66421 Homburg/Saar, Germany; Department of Anatomy II, Friedrich Alexander University Erlangen-Nürnberg, Erlangen, Germany

## Abstract

**Background:**

Surfactant proteins (SP) secreted by alveolar type 2 cells, play an essential role in maintaining the air-liquid barrier of the lung and are also involved in the opsonisation and clearance of bacteria by phagocytes. We have recently described a novel surfactant protein, SP-H (SFTA3). Expression of SP-H was earlier demonstrated to be upregulated by LPS and negatively regulated by IL-1β and IL-23 *in vitro*. The influence of SP-H on phagocytosis was measured using a murine and a human phagocytic cell line and fluorescent latex beads.

**Findings:**

SP-H markedly increases phagocytosis *in vitro* in the murine-derived alveolar macrophage cell lines MH-S and in human-derived differentiated U937 cells.

**Conclusion:**

It can be assumed that SP-H is involved in regulating phagocytic activity of macrophages. SP-H is a new player in pulmonary host defence.

## Background

Pulmonary surfactant proteins (SP) reduce the surface tension at the air-liquid interface and act as collectins in innate immunity. The lack of SPs leads to respiratory failure. SP-A and SP-D bind to carbohydrates of bacteria, viruses, fungi and protozoa, opsonize them and thereby accelerate their removal by phagocytes [1, 2]. The role of SP-A in opsonisation and removal of *Staphylococcus aureus* is well-known and was recently confirmed *in vivo* by the decelerated phagocytosis of *S. aureus* in SP-A deficient mice [3], although it has recently been shown that *S. aureus* express and secrete SPs as well [4]. Surfactant proteins B and C are involved in the stabilization of phospholipids in the air-liquid interface [5]. In addition to the four known surfactant proteins SP-A, SP-B, SP-C and SP-D [6, 7], a novel surfactant protein called SFAT3 or SP-H was recently identified by Schicht *et al.* in lung tissues [7]. This protein, with a molecular weight of 10 kDa, was first detected by means of bioinformatics and subsequently identified in human lung and bronchoalveolar lavage at concentrations of 0.06–1.83 ng mg^-1^ and 0.15–4.87 ng mg^-1^. Within lung tissue, SP-H is distributed in alveolar type 1 and 2 cells, alveolar macrophages and in the cytoplasm of the epithelium. Using the alveolar cell line A549, an increase in SP-H expression was demonstrated as a response to cell stimulation with bacterial lipopolysaccharide (LPS), *in vitro*. In contrast, interleukin (IL)-1β and IL-23 caused the down regulation of the SP-H mRNA in A549 cells. This observation led the authors to the assumption that SP-H might play a role in host defence against gram negative bacteria [8]. In this study, we show the phagocytosis efficiency increasing property of SP-H.

## Findings

For this purpose, we have used recombinant SP-H (GST purification) and an in vitro phagocytosis assay as previously described [8, 9]. The murine alveolar macrophages cell line MH-S was obtained from Sigma-Aldrich (Germany). Cells were grown in RPMI 1640 medium (Lonza, Germany) containing 10% heat inactivated fetal bovine serum (PAA, Germany), 100 Units ml^-1^ penicillin, 100 μg ml^-1^ streptomycin (both antibiotics from PAA, Germany) and 50 μM β-mercaptoethanol (gibco life technologies, Darmstadt, Germany) at 37°C in a humidified atmosphere with 5% CO_2_. To detach the cells from culture flasks a few milliliters of a mixture of 500 μg ml^-1^ trypsin with 220 μg ml^-1^ EDTA were added to the adherent cells, which were then incubated at 37°C for fifteen minutes. Cells were then collected by centrifugation and resuspended in medium. Then 0.5 × 10^6^ or 1 × 10^6^ cells were disseminated on 12 well-plates (Greiner Bio-One, Frickenhausen, Germany). After 15 hours the cells were used for the phagocytosis assay. The monocyte-like human lymphoma U937 cell line was cultivated in RPMI 1640 medium with 10% fetal bovine serum, 100 Units ml^-1^ penicillin and 100 μg ml^-1^ streptomycin. For differentiation into adherent macrophage-like cells capable of phagocytosis, cells were incubated with 50 ng ml^-1^ phorbol 12-myristate 13-acetate (PMA) (Sigma Aldrich) for 20 hours, washed with medium once, and then incubated in medium for 48 hours at 37°C in a humidified atmosphere with 5% CO_2_. Subsequently the cells were used for the phagocytosis assay. Fluoresbrite® Yellow Green Microspheres (Polysciences GmbH, Eppenheim, Germany) with a size of 1 μm were used for phagocytosis assays in presence of 0, 100 ng ml^-1^, 250 ng ml^-1^, 500 ng ml^-1^ and 1000 ng ml^-1^ SP-H. After 120 min incubation, the cells were washed and the percentage of the cells with ingested fluorescent beads was determined using a flow cytometer (FACS Calibur, Beckton Dickinson, Heidelberg, Germany). The assays were repeated twice with SP-H expressed in *E.coli* (n = 2) and reproduced with SP-H expressed in mammalian cells (n = 2) (data not shown). The statistical significance (P < 0.05) was analysed by ANOVA, Dunnett’s Multiple Comparison test and Bonferronis Multiple Comparison test. For the first posthoc test, the SP-H free cells served as reference values. To determine the specifity of SP-H for the phagocytosis, experiments were also conducted after incubating the cells with three inhibitors, cytochalasin D (5 mg ml^-1^), nocodazol (3 mg ml^-1^) and staurosporine (10 μM), previously revealed to inhibit phagocytosis significantly [9].

**Figure 1:**
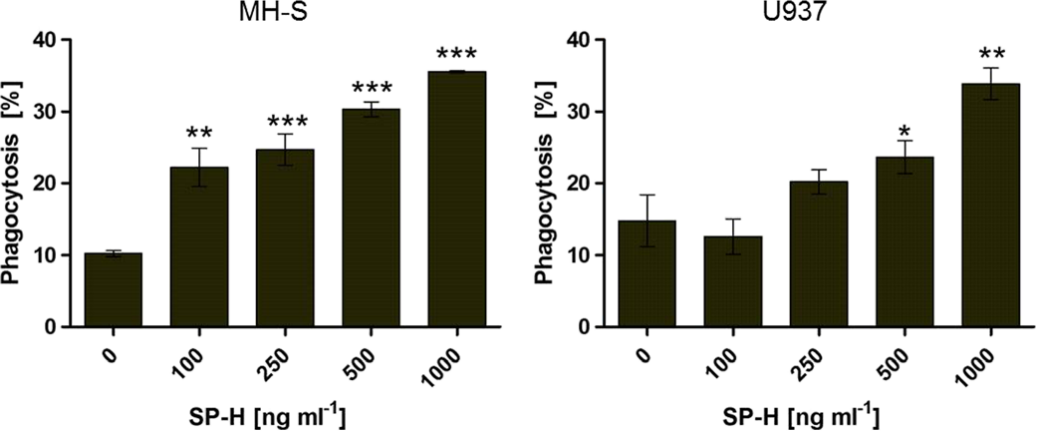
**The phagocytosis efficiency of the alveolar macrophage cell line MH-S and the differentiated lymphoma cell line U937 as a function of SP-H.** The phagocytosis efficiency was determined by flow cytometry.

**Figure 2:**
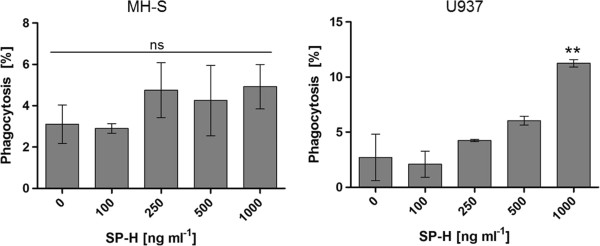
**The phagocytosis efficiency of the alveolar macrophage cell line MH-S and the differentiated lymphoma cell line U937 as a function of SP-H after incubation in a mixture of three phagocytosis inhibitors: cytochalasin D**, **nocodazole**, **and staurosporine.** Ns: not significant. The phagocytosis efficiency was determined by flow cytometry.

**Figure 3:**
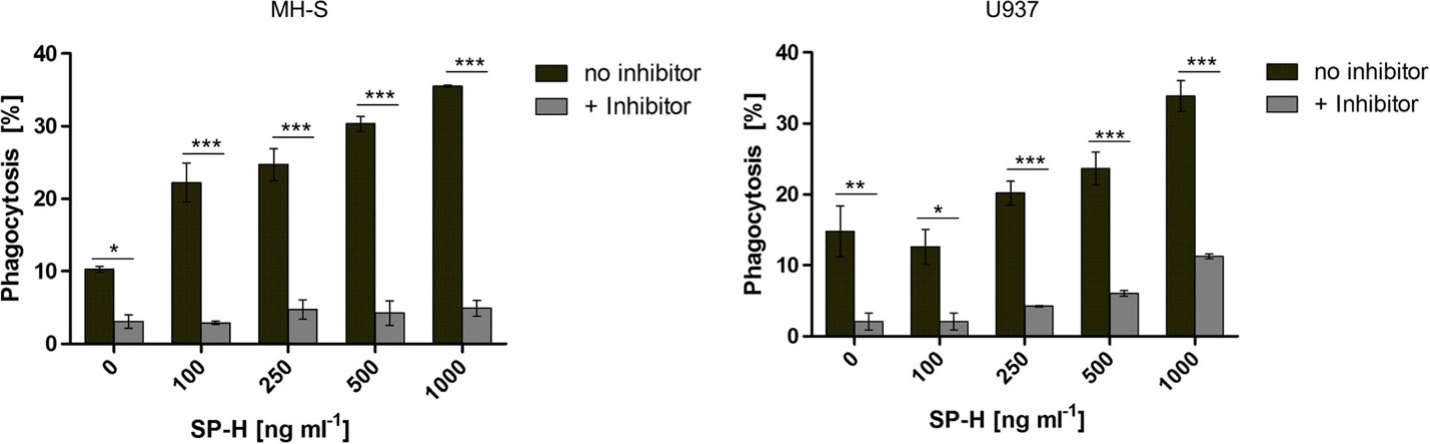
**Comparison of phagocytosis efficiencies in absence and presence of phagocytosis inhibitors cytochalasin D,** **nocodazole and staurosporine.** The data shows that treatment of the cells with a mixture of the inhibitors reduces phagocytosis efficiency significantly.

The results revealed that the efficiency of bead uptake by both cell lines was significantly enhanced by the presence of the SP-H protein. The gradually increasing concentration of the protein also caused a gradual increase in phagocytosis efficiency. The results show that, in the presence of 500 ng ml^-1^ and 1 μg ml^-1^ SP-H, both cell lines are significantly stimulated to take up particles (Figure 1). Obviously, the murine alveolar macrophage cell line MH-S has a higher susceptibility to SP-H than the human U937, which was initially isolated from histocytic lymphoma [10]. In summary, the findings of this study demonstrate an improving effect of SP-H on the phagocytosis of latex particles by macrophage like cell lines of human and of mouse origin. Especially the alveolar macrophage cell line MH-S is highly stimulated by SP-H, even at a concentration of 100 ng ml^-1^, whereas the differentiated U937 cells showed only slightly increased phagocytosis efficiency at a five-fold higher concentration (500 ng ml^-1^). To exclude that an increase in the relative fluorescence intensity (RFI) measured by flow cytometer is caused by an enhanced adherence of the polystyrene microspheres to the cells, the assays were performed after incubation of the cells with phagocytosis inhibitors. Figure 2 shows that in the presence of the inhibitors, SP-H did not have a phagocytosis efficiency increasing effect on MH-S cells. In contrast, U937 cells treated with phagocytosis inhibitors, showed a significant increase in their phagocytosis efficiency caused by 1 μg ml^-1^ SP-H. However, the phagocytosis of the U937 cells with 1 μg ml^-1^ SP-H treated with inhibitors is significantly lower than the U937 cells that were not treated with inhibitors (Figure 3). Therefore, the increase in RFI values are attributable to an enhanced phagocytosis efficiency, rather than to improved adhesion of the beads to the cells.

Based on these results, we assume that one *natural* role of SP-H is to enhance alveolar macrophage phagocytosis. SP-H enhances the phagocytosis efficiency of the human-derived and the murine-derived macrophage cell lines for polystrene microspheres. This suggests that SP-H could be involved in foreign particle clearance and possibly into pathogen clearance *in vivo.*

## Abbreviations

A549: Adenocarcinomic human alveolar basal epithelial cells
ANOVA: Analysis of variance
GST: Gluthatione S-transferase
IL: Interleukin
LPS: Lipopolysaccharide
MH-S: Murine alveolar macrophage cell line
SFTA3: Surfactant Associated 3
SP (A: B: C, D, H), Surfactant Protein (A, B, C, D, H)
U937: Human histiocytic lymphoma-derived cell line
PMA: Phorbol 12-myristate 13-acetate.
